# Flow and wall shear stress in end-to-side and side-to-side anastomosis of venous coronary artery bypass grafts

**DOI:** 10.1186/1475-925X-6-35

**Published:** 2007-09-26

**Authors:** Thomas Frauenfelder, Evangelos Boutsianis, Thomas Schertler, Lars Husmann, Sebastian Leschka, Dimos Poulikakos, Borut Marincek, Hatem Alkadhi

**Affiliations:** 1 Institute of Diagnostic Radiology, University Hospital Zurich, Zurich, Switzerland; 2 Laboratory of Thermodynamics in Emerging Technologies, ETH Zurich, Zurich, Switzerland

## Abstract

**Purpose:**

Coronary artery bypass graft (CABG) surgery represents the standard treatment of advanced coronary artery disease. Two major types of anastomosis exist to connect the graft to the coronary artery, i.e., by using an end-to-side or a side-to-side anastomosis. There is still controversy because of the differences in the patency rates of the two types of anastomosis. The purpose of this paper is to non-invasively quantify hemodynamic parameters, such as mass flow and wall shear stress (WSS), in end-to-side and side-to-side anastomoses of patients with CABG using computational fluid dynamics (CFD).

**Methods:**

One patient with saphenous CABG and end-to-side anastomosis and one patient with saphenous CABG and side-to-side anastomosis underwent 16-detector row computed tomography (CT). Geometric models of coronary arteries and bypasses were reconstructed for CFD analysis. Blood flow was considered pulsatile, laminar, incompressible and Newtonian. Peri-anastomotic mass flow and WSS were quantified and flow patterns visualized.

**Results:**

CFD analysis based on in-vivo CT coronary angiography data was feasible in both patients. For both types of CABG, flow patterns were characterized by a retrograde flow into the native coronary artery. WSS variations were found in both anastomoses types, with highest WSS values at the heel and lowest WSS values at the floor of the end-to-side anastomosis. In contrast, the highest WSS values of the side-to-side anastomosis configuration were found in stenotic vessel segments and not in the close vicinity of the anastomosis. Flow stagnation zones were found in end-to-side but not in side-to-side anastomosis, the latter also demonstrating a smoother stream division throughout the cardiac cycle.

**Conclusion:**

CFD analysis of venous CABG based on in-vivo CT datasets in patients was feasible producing qualitative and quantitative information on mass flow and WSS. Differences were found between the two types of anastomosis warranting further systematic application of the presented methodology on multiple patient datasets.

## Background

### Medical investigations

Coronary artery bypass graft (CABG) surgery represents the standard treatment of advanced coronary artery disease (CAD). Since the pioneering work of Favaloro [1], various grafts and different surgical techniques have been investigated. Regarding anastomoses, two major types exist to connect the graft to the coronary artery, i.e. by using an end-to-side or a side-to-side anastomosis. Although the latter has been reported to provide some advantages over individual grafting with end-to-side anastomosis [2-4], the sequential grafting technique has often been criticized because of differences in the patency rates of the two types of anastomosis [2,5,6]. The long-term clinical outcome after myocardial revascularization is dependent on graft patency. In venous CABG, however, accelerated atherosclerosis has been repetitively reported [7]. During the first year after CABG surgery up to 15% of venous grafts occlude, between 1 and 6 years the graft attrition rate is 1% to 2% per year, and between 6 to 10 years it is 4% per year. By 10 years after surgery only 60% of vein grafts are patent and only 50% of patent vein grafts are free of significant stenosis [8].

Neointimal hyperplasia, defined as the accumulation of smooth muscle cells and extracellular matrix in the intimal compartment, is the major disease process in venous CABG in the first year after surgery and sets the foundations for later development of graft atheroma [7]. In support of this proposal, the American Heart Association Council on Arteriosclerosis has defined the localized areas of adaptive neointimal hyperplasia as atherosclerosis prone regions [9]. During the first month after CABG surgery vein graft attrition results from thrombotic occlusion [10], while later on the dominant process is atherosclerotic obstruction occurring on a foundation of neointimal hyperplasia [11]. Although the risk factors predisposing to vein graft atherosclerosis are broadly similar to those recognized for native CAD, the pathogenic effects of these risk factors are amplified by inherent deficiencies of the vein as a conduit when transposed into the coronary arterial circulation [7].

In attempts to prevent acute and late graft occlusion, much effort has been invested in identifying the etiology of anastomotic neointimal hyperplasia and the plausibility of its prevention. Hypotheses related to this subject include the concept of compliance mismatch between graft and host artery [12], high frequency flow and wall shear stress (WSS) [13] as well as abnormal flow dynamics at the distal anastomosis [14]. Sottiurai et al. [15] have compared in an animal model the development of neointimal hyperplasia in end-to-side versus side-to-side anastomoses. Their study revealed that neointimal hyperplasia was present at the heel, toe, and floor of the end-to-side but not in the side-to-side anastomoses. Since compliance mismatch was not at issue in their model by using autogenous femorofemoral bypass grafts, the geometry of the distal anastomosis was attributed to be the causal factor. The configuration of end-to-side anastomosis is not a common occurrence in the primate cardiovascular system except for a patent "ductus arteriosus Botalli" [16].

Hemodynamic patterns at distal CABG anastomoses are thought to exhibit flow separation, recirculation and moving stagnation zones. Such flow features correspond typically to low time averaged WSS, long residence times, shear oscillation and increased spatial WSS gradients. These are the main hemodynamic features that have been connected with atherogenesis and intimal hyperplasia [17,18]. In a recent review Kassab and Navia [19] put forward the homeostasis hypothesis as the underlying mechanism of graft failure. It is postulated that there is a mechanical homeostatic state for each blood vessel. Growth and remodeling takes place to abate perturbations from this state, such as when a vein segment is used in CABG. The remodeling procedure can fail or lead to a non-physiological state when the incurred perturbations are sufficiently large. Hemodynamic flow assessment in coronary arteries and CABGs is commonly performed with intravascular Doppler ultrasound [20]. However, this approach is invasive and the introduction of an ultrasound-catheter leads to flow disturbances thus interfering with the measurements [21]. Lately, phase-contrast magnetic resonance imaging (PC-MRI) [22] provides a non-invasive means of in-vivo three dimensional instantaneous velocity measurements on selected arterial cross-sections at a sufficient resolution.

### CFD investigations

An alternative to invasive or non-invasive flow measurements is the simulation of blood flow by using computational fluid dynamics (CFD) and/or ex-vivo experimental flow setups. Numerical studies on physiological coronary flow as well as on CABG flow have been published extensively during the last 15 years. Steinman [23] presented a transient two dimensional flow simulation within a rigid 45 degree end-to-side anastomosis model. His results indicated elevated instantaneous WSS values at the toe and heel of the graft-host junction as well as along the host artery bed. In 1998 Ethier [24] simulated three dimensional unsteady flows in a symmetrical 45 degree end-to-side configuration with solid walls. It was shown that the perianastomotic WSS distributions are influenced by a complex interplay between secondary flow effects and the unsteadiness of the graft inflow waveform. An authoritative numerical description of the flow separation patterns of 45 degree junctions was given in [25] under steady flow conditions for a wide range of inlet Reynolds (Re) numbers, 250 – 1650. This study utilized an adaptive mesh refinement scheme to ensure grid independency.

Research on the hemodynamics of anastomoses has largely focused on determining the influence of the geometric characteristics of end-to-side configurations on WSS distributions. Fei [26] presented a series of steady flow simulations for a set of anastomosis angles ranging from 20 to 70 degrees. More recently Freshwater [27] utilized pulsatile flow boundary waveforms typical of a left internal mammary artery graft to the left anterior descending coronary artery scenario. They showed that higher anastomotic angles result in higher WSS magnitude values and flow oscillation around the toe and along the bed of the host branch. In all of the studies referenced so far, the centerlines of the graft and the host vessel lie within the same plane therefore forming planar configurations. Sherwin [28] investigated the influence of out of plane geometry in stiff end-to-side anastomosis models under steady and Papaharilaou [29] under transient flow conditions. Such configurations are closer to reality and result in non-symmetric flow fields. In general, the anastomosis bed was affected the most with reduced peak WSS values in comparison to similar planar models, while the mean oscillatory WSS magnitude was also shown to decrease. In most of the aforementioned investigations the native coronary or host vessel was fully occluded. Several researchers have addressed the issue of prograde and/or retrograde flow through the native coronary artery, as in [30,31] under steady flow conditions and in [32] for pulsatile flow. In order to assess the potential role of activated platelets in the pathogenesis of intimal hyperplasia and/or thrombogenesis, numerical models have tried to quantify shear exposure and near wall residence times [33,34]. Finally, Sankaranarayanan [35,36] presented two studies, in a planar and a non-planar three dimensional CABG model respectively, that included the proximal anastomosis to the aorta.

It is worth noticing that most of the published CFD studies on anastomosis hemodynamics have been evaluated versus equivalent flow experiments by utilizing a variety of flow visualization, velocity and WSS measurement techniques such as in [37-40]. Combined investigations utilizing CFD and PC-MRI on coronary and bypass flows are of particular interest because of the potential of MRI in providing in-vivo velocity measurements as well as anatomical information [41]. The vast majority of the above mentioned investigations focused on the hemodynamics of end-to-side anastomosis configurations. Bonert [42] examined the hemodynamics of side-to-side CABG anastomosis in solid idealized geometric models. They concluded that the parallel form of side-to-side anastomosis, as opposed to a non-parallel one, is better suited to maintain graft patency. A comparison between side-to-side and end-to-side anastomosis showed increased hemodynamic risk in the former approach due to the presence of larger low WSS areas.

### Purpose of the present investigation

Advances in the CFD software and its corresponding hardware along with medical imaging are leading to the generation of increasingly reliable computational models. We can retrace a similar development into the fluid dynamics investigations of physiological coronary flow. Intracoronary flow can now be addressed in anatomically accurate configurations ranging from stiff multi-branched models [43] to moving and compliant arterial sections [44]. The present study investigated hemodynamic features of CABG anastomoses using patient-specific data. Pulsatile blood flow in venous CABGs was simulated using CFD on solid patient-specific geometric models based on in-vivo CT coronary angiography datasets. We assessed differences in WSS, mass flow, and flow patterns in venous conduits with end-to-side compared to side-to-side anastomosis.

## Methods

### Patients

Two male patients, 64 and 55-years old respectively, underwent cardiac CT. Patient 1 suffered from CAD with recurrent episodes of angina pectoris and a recent myocardial infarction. Invasive coronary angiography revealed a significant stenosis of the proximal right coronary artery (RCA) and non-significant stenoses of the middle left anterior descending (LAD) and distal left circumflex artery (LCX). Subsequently, the patient underwent saphenous CABG surgery with an end-to-side anastomosis onto the distal RCA. Patient 2 suffered from dyspnoea and instable angina pectoris. Invasive coronary angiography showed serial significant stenoses of the proximal LAD and a significant stenosis of the distal LCX. Saphenous CABG surgery was performed with a sequential side-to-side anastomosis onto the middle LAD and an end-to-side anastomosis onto the distal LCX.

### CT data acquisition

CT was performed 10 and 15 days after surgery, respectively, on a 16-detector row scanner (Sensation 16, Siemens Medical Solutions, Forchheim, Germany) using the following parameters: detector collimation 16 × 0.75 mm, gantry rotation time 0.37 sec, pitch 0.38, tube potential 120 kV, tube current time product 400 mAs. A bolus of 150 ml iodinated contrast material (iodixanol, Visipaque 320, 320 mg/ml, GE Healthcare, Buckinghamshire, UK) followed by 30 ml saline solution was continuously injected into a right antecubital vein via a 18-gauge catheter at a flow rate of 5 ml/sec. Bolus tracking was performed with a region of interest in the ascending aorta and image acquisition was automatically started 5 sec after signal attenuation reached a threshold of 140 HU. Synchronized to the electrocardiogram (ECG), CT data sets were retrospectively reconstructed throughout the cardiac cycle in 5% steps of the R-R interval with a slice thickness of 1 mm and an increment of 0.5 mm using a medium soft-tissue convolution kernel (B30f). The adaptive cardio volume approach was used for image reconstruction and ECG-pulsing was applied to reduce radiation exposure. The reconstruction phase providing best image quality with the lowest degree of motion artifacts was determined by two readers in consensus and was used for further post-processing. The local ethics committee approved the study protocol and written informed consent was obtained from both patients.

### Geometric reconstruction

**Figure 1:**
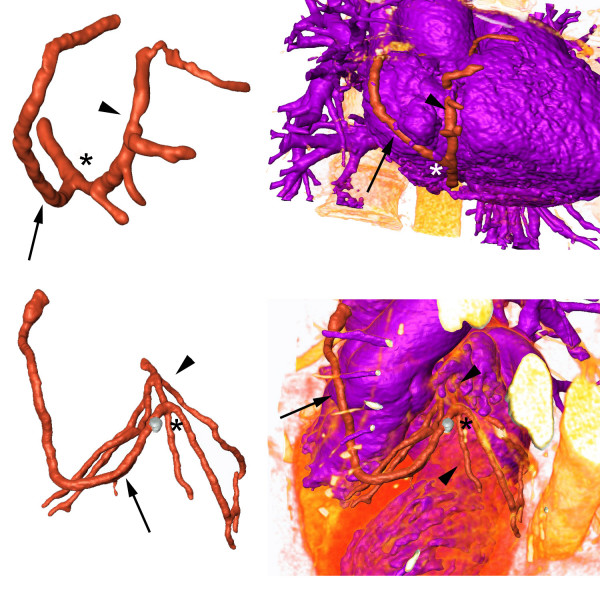
**Models**. Anatomical depiction of the two models including the aortocoronary bypasses (arrows), the coronary arteries (arrowheads), the anastomoses (asterisks) and their relation to the heart and the aorta.

Axial CT images were digitally processed to extract geometrical contours representing the coronary arteries and the CABGs. The lumen of all coronary arteries and grafts of the two patients were semi-automatically segmented using a commercially available software package (Amira 3.1, TGS, Belgium). In regions of reduced arterial opacification, segmentation was manually complemented. The outflows (i.e., the end of the branches) and inflows (i.e., the ostia) of the vessels were separately marked to allow the imposition of boundary conditions. As a next step, an unstructured surface mesh of triangles was generated covering the segmented volume using the marching cube algorithm. Manual smoothing and low-pass spatial filtering was then applied to further reduce fine-scale surface irregularities. The final model depicted the real three dimensional (3D) geometry of the coronary arteries and bypass grafts (Figure 1). Four computational models, two for each case, were subsequently built with 750.000 – 900.000 tetrahedral cells. The finest meshes represented a spatial resolution of about 0.15 mm and the number of elements per cross-section ranged from 150 to 200 depending on the vessel diameter.

### Model assumptions and boundary conditions

**Figure 2:**
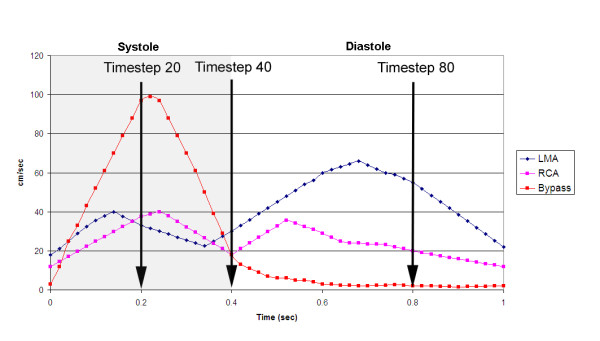
**Input functions**. Flow velocities throughout one cardiac cycle used as input functions at the ostium of the left main artery (LMA), right coronary artery (RCA), and the coronary artery bypass graft.

The flow for the simulation was considered transient, 3D, incompressible, and laminar. Corresponding to standard values from the literature, blood was assumed Newtonian with a viscosity of 0.0037 Pa· sec and a density of 1060 kg/m^3^. The walls were modeled as solid and stiff and a zero-velocity boundary condition was adopted at the fluid-solid interface, corresponding to a no-slip condition. In contrast to other flow simulations, we chose not to elongate the inlet part for the coronary arteries to allow for a full flow development. The instantaneous velocity at the different inlets (coronary ostia and proximal bypass anastomoses) was based on standard data [45,46] reflecting the physiologically pulsatile, biphasic blood velocity from the ascending aorta into the coronary arteries (Figure 2). We adopted a spatially uniform profile for these boundary cross-sections. The maximum inlet Re was calculated at 1230 (bypass inlet) lasting for a very short period of time. The mean Re ranged between 380 (right coronary artey inlet) and 570 (left coronary artery inlet). The duration of the cardiac cycle was normalized to 1 second for both patients. The corresponding Womersley (Wo) number was 3.5 (bypass inlet), 2.1 (right coronary inlet) and 2.45 (left coronary inlet). The stress-free boundary condition (zero normal and tangential stresses), which arises naturally from the application of the finite element method, was imposed on the velocity field at all outlets to facilitate a common comparison frame between these two highly different coronary circulations by removing the influence of their downstream impedances. In this way, the observed flow differences are mainly the result of the adopted anastomosis type.

### Computational fluid dynamics

The finite element software FIDAP (Version 8.6.2, Fluent Corp., Darmstadt, Germany) was used to perform the CFD simulations by solving the Navier-Stokes equations with linear basis functions. Calculated flow variables were flow velocities and pressure following a segregated solution approach. A convergence criterion of four orders of magnitude was adopted for the residuals in velocity and pressure. The instantaneous flow field was acquired at 100 steps per cardiac cycle with a constant time step using backward Euler implicit time integration. All the results presented herein belong to the third computational cardiac cycle to allow ample time for the attenuation of the effects of the initial conditions. The sensitivity of the numerical results on the underlying grid was examined under steady flow conditions. The time averaged values of the inflow velocities, shown in Figure 2, were used for this purpose by adopting a spatially uniform profile. The resulting differences in the calculated velocity values averaged below 5% of the maximum prescribed inflow velocity. This was judged sufficient for the requirements of the present feasibility investigation. The presented CFD results for both patients came from the finest available meshes. The Fieldview software (Version 11.0, Intelligent Light, Lyndhurst, NJ) was used for visualization of flow patterns, quantification of WSS and volumetric fluxes at selected sites.

## Results

Mean heart rate during CT scanning was 47 ± 9 bpm for patient 1 and 51 ± 6 bpm for patient 2. The percent phase providing best image quality was found between 50 and 60% of the RR-interval for the RCA and between 60 and 70% for the left main artery (LMA), LAD, and LCX.

### End-to-side anastomosis

#### Mass flow

**Figure 3:**
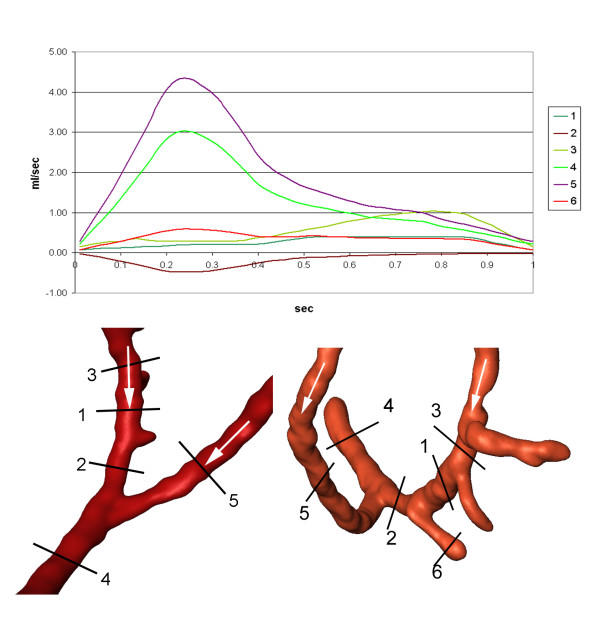
**Flux in end-to-side anastomosis**. Integrated volumetric flux (ml/sec) at indicated cross-sections (cuts) in the RCA and the end-to-side anastomosis.

**Figure 4:**
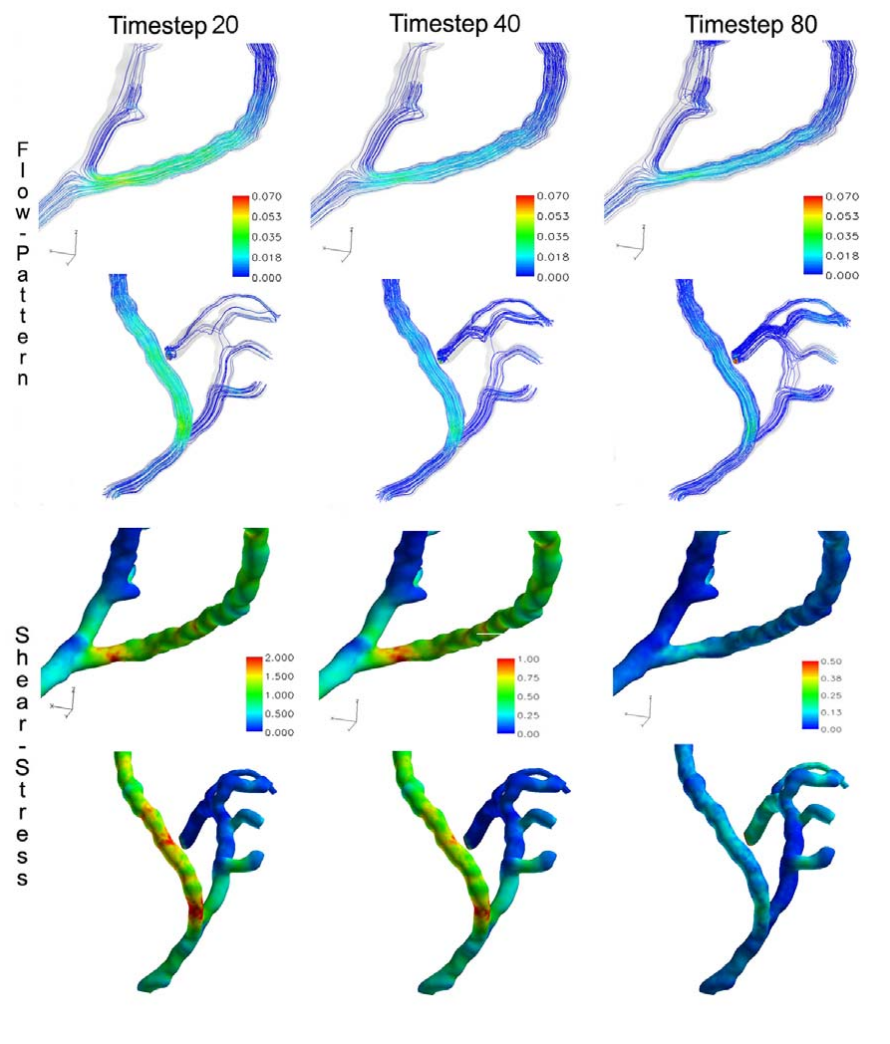
**Velocity and shear stress in end-to-side anastomosis**. Velocity-coded streamlines (m/sec) and wall shear stress (Pa) in an end-to-side anastomosis at three different time steps of the cardiac cycle.

The mean volumetric flow through the right coronary artery was 3.07 ml/sec. The mean volumetric flow through the CABG was 1.81 ml/sec (Figure 3; cut 5). Quantifications of mass flow through the proximal and distal part of the right coronary artery and the bypass close to the end-to-side anastomosis are demonstrated in Figure 3. The distribution of pulsatile flow could be determined by using the flow curves at each time-step of the cardiac cycle. There was a significant backflow into the proximal segment of the RCA (Figure 3; cut 2), reaching a maximum of 0.48 ml/sec. Due to this high backflow, the next proximal branch of the RCA (Figure 3; cut 4) was filled by blood coming from the bypass for the largest part of the cardiac cycle. The amount of backflow depends on the coronary mass flow and the bypass mass flow, which are characterized by different mass flow curves at their inlets, the angle between the bypass and the RCA, and the degree of the stenosis in the bypassed artery. The retrograde flow into the RCA reached a distance of 2.8 cm from the end-to-side anastomosis (Figure 4), leading to an area of stagnation between the two more proximal branches (Figure 3; cut 1 and cut 2).

#### Flow patterns and WSS

**Figure 5:**
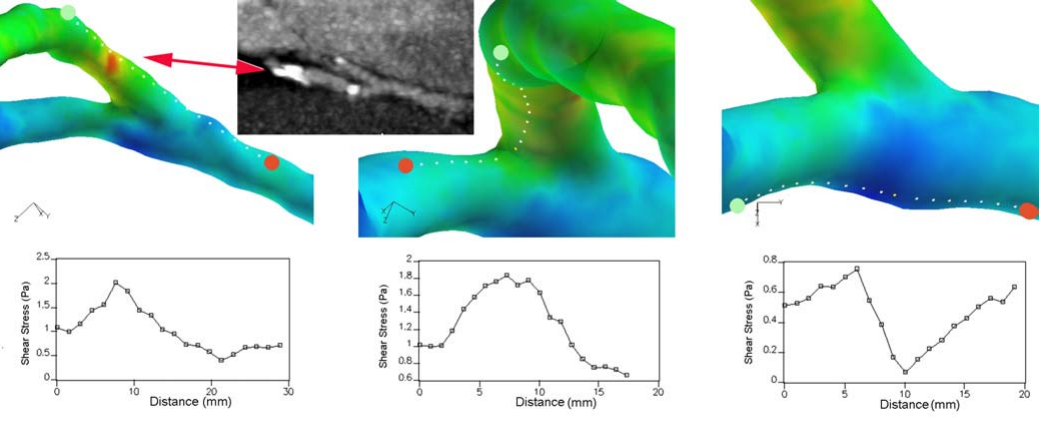
**Shear stress distribution in end-to-side anastomosis**. Color-coded wall shear stress (Pa) and corresponding values plotted against the distance (mm) from the green to the red point at timestep 20 on three different paths upon the walls of the end-to-side anastomosis.

The WSS characterizes the tangential fluid forces that act on the vessel wall. The changes of WSS throughout the cardiac cycle showed a correlation with flow velocities, with WSS forces being high when blood flow was fast. Near the end-to-side anastomosis the maximum WSS spatial variation was approximately 1.5 Pa (Figures 4 and 5). The WSS ranged from 0.01 Pa at minimum inflow to approximately 2.0 Pa at maximum inflow. In the perianastomotic region, the time averaged WSS was 0.36 Pa. An area of high WSS was found during systole at the heel of the anastomosis. The lowest WSS was found in the RCA at the location where blood from the CABG hit the wall and was confronted by the flow coming from the native coronary artery. This formed a stagnation point at the impact site around which the bypass stream splits, as depicted by the flow streamlines in Figure 4. It is also shown here that backflow into the RCA persisted during most of the cardiac cycle. The highest WSS values were found during mid-systole (timestep 20) and were located at the heel of the anastomosis and at the side of a nearby clip that was placed during surgery (Figure 5). At the opposite side of the anastomosis the WSS values were lower, due to the presence of the stagnation zone.

### Side-to-side anastomosis

#### Mass flow

**Figure 6:**
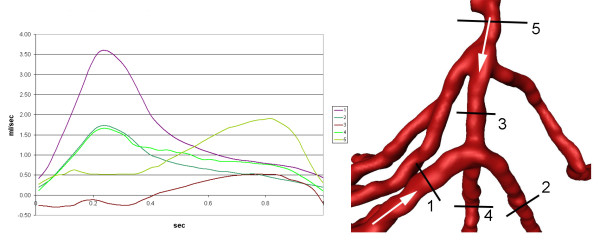
**Flux in side-to-side anastomosis**. Integrated volumetric flux (ml/sec) at indicated cross-sections (cuts) in the LAD and the side-to-side anastomosis.

The mean volumetric flow through the left coronary artery was 3.35 ml/sec. The mean volumetric flow through the CABG was 1.51 ml/sec. Quantifications of volumetric flow through the proximal and distal segment of the LAD and the proximal and distal bypass close to the side-to-side anastomosis are demonstrated in Figure 6. The distribution of pulsatile flow could be determined by using the flow curves at each time-step of the cardiac cycle. There was a small backflow into the proximal part of the left coronary artery (Figure 6; cut 3) lasting almost 40% of the cardiac cycle and reaching a maximum of 0.25 ml/sec. The maximum volumetric flux into the distal part of the artery (Figure 6; cut 4) was 1.65 ml/sec or 46.2 % of the entire flow. Throughout a cardiac cycle, 53% of the mass flow into the bypass remained in it and reached the next anastomosis (Figure 6; cut 2). In diastole additional blood circulated from the proximal into the distal part of the coronary artery (Figure 6; cut 3), partially compensating for the decrease in the amount of blood that came from the bypass (Figure 6; cut 4).

#### Flow patterns and WSS

**Figure 7:**
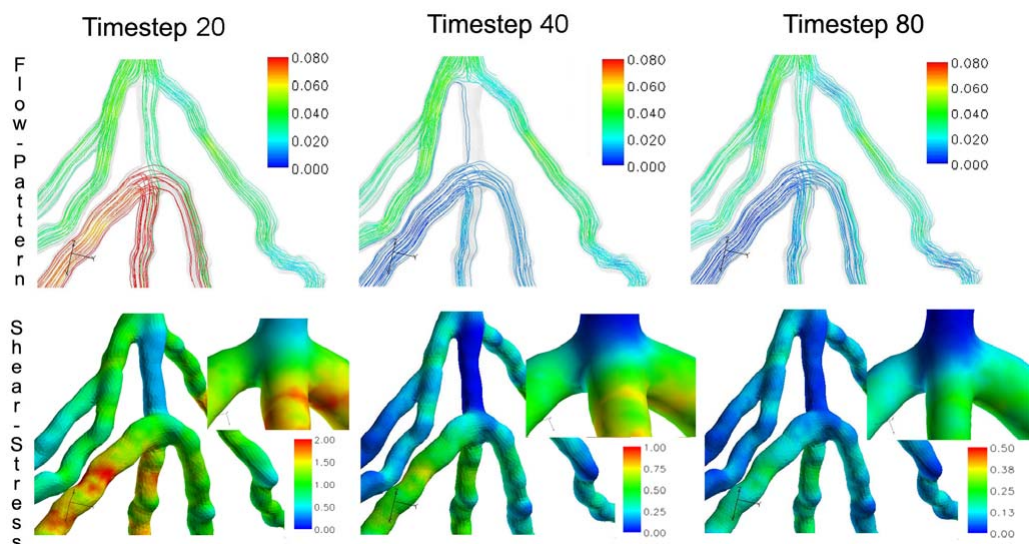
**Velocity and shear-stress in side-to-side anastomosis**. Velocity-coded streamlines (m/sec) and wall shear stress (Pa) in a side-to-side anastomosis at three different time steps of the cardiac cycle.

**Figure 8:**
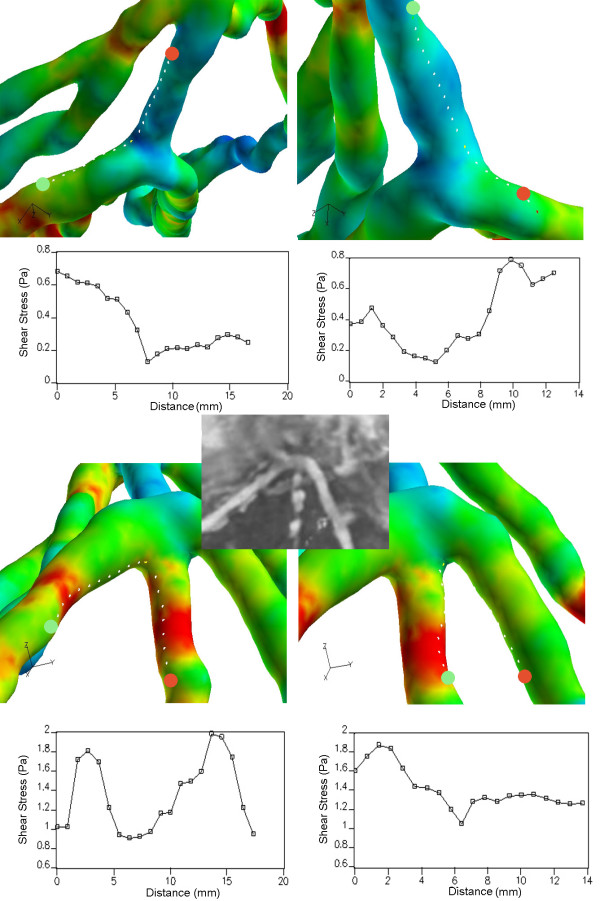
**Shear stress distribution in side-to-side anastomosis**. Color-coded wall shear stress (Pa) and corresponding values plotted against the distance (mm) from the green to the red point at timestep 20 on four different paths upon the walls of the side-to-side anastomosis.

Comparing side-to-side to end-to-side anastomosis, the mean WSS values were lower in the side-to-side anastomosis with a time-averaged value of 0.29 Pa in the perianastomotic region (Figure 7 and 8). The WSS values ranged from 0.02 Pa up to approximately 2.1 Pa at mid-systole. There were some areas of elevated WSS close to the anastomosis, but the highest WSS values were found in the atherosclerotic distal coronary artery (Figure 8). The elevated WSS in the distal coronary artery was mainly caused by increased bypass mass flow during systole. Furthermore, there was a high WSS region in the proximal part of the bypass due to a mild stenosis (Figure 7 and 8). In contrast, in the end-to-side anastomosis highest WSS values were located in the distal bypass graft close to the anastomosis. Lowest WSS values were found in the native coronary artery proximal to the side-to-side anastomosis. The maximum WSS spatial variation was approximately 1.0 Pa, being smaller than the WSS variation in end-to-side anastomosis. The streamline patterns presented in Figure 7 indicate absence of stagnation zones in the perianastomotic region of side-to-side anastomosis and a smoother stream division throughout the cardiac cycle as compared to the end-to-side anastomosis.

## Discussion

The results from our study indicate that considerable variations of blood flow exist in the distal perianastomotic region of venous CABG that may coincide with areas of neointimal hyperplasia. We found WSS variations in both types of anastomoses, with highest WSS values at the heel and lowest WSS values at the floor of the end-to-side anastomosis case. In contrast, high WSS in the side-to-side anastomosis configuration was only found in stenotic vessel segments but not in the close vicinity of the anastomosis. Across both types of anastomoses, elevated WSS values did coincide with vessel stenosis, being either caused by atherosclerotic wall changes or by a surgical clip.

CFD provided detailed information on instantaneous mass flow. In the case of side-to-side anastomosis, this information was important to predict the mass flow reaching the next anastomosis. In addition, the amount of blood running retrograde in the coronary artery and eventually reaching a more proximal branch can be quantitatively measured. The numerical results in our patients revealed considerable retrograde flow into the host coronary artery in both types of anastomoses, a finding that has been previously described in experimental models [38]. On the other hand stagnation zones were only found in end-to-side as compared to side-to-side anastomosis. The latter configuration showed smoother stream divisions of mass flow throughout the cardiac cycle. Finally, it was clearly demonstrated that the instantaneous blood influx through the bypasses maximizes during systole. Conversely, under physiological coronary flow conditions the maximum flux into the system occurs during diastole. This is a major difference that leads to a more balanced total instantaneous flow in the cases reported herein. Possible pathophysiological implications of this fact warrant further research.

When investigating hemodynamic features of blood flow, numerical simulation has become an important tool. This study presents realistic, patient-specific models based on CT angiography datasets of coronary and CABG anatomy. This approach circumvents the need for idealization of the main geometrical features. Most of the CFD studies that were mentioned in the introduction had to rely on some sort of geometrical regularization by introducing pre-specified parameters such as the CABG angle, the host to graft diameter ratio and the planarity or non-planarity of the entire configuration. There are merits in the latter methodology. There is the ability to parametrically study the hemodynamic effects of these strictly defined morphological parameters. Furthermore the generation of the underlying surface and volumetric grids is much easier and can lead to numerical results of greater accuracy. On the other hand, a patient specific model is much more difficult to construct and to ensure sufficient numerical accuracy. It is a closer match to reality but at the same time it is harder to parameterize the results and extract generalized conclusions.

CT angiography currently provides the most accurate representation of real anatomy. In contrast to most computational studies, our geometric models did not only include a small part of a coronary artery or CABG bypass but entailed the coronary tree and bypass grafts from their origin to the distal anastomoses. This became possible with the improvement of multi-detector row CT scanner technology with fast gantry rotation times enabling accurate and reliable depiction of coronary and bypass graft anatomy in a short imaging time [47,48]. With the advent of 64-slice CT [49] and dual-source CT [50] further improvements with regard to temporal and spatial resolution have been made allowing an even more precise depiction of anatomy that will further improve the accuracy of numerical flow simulations.

The following study limitations have to be acknowledged. Firstly, 16-detector row CT does not offer currently the best imaging for coronary arteries and CABG as compared to newer scanner technology. In addition, geometric data for CFD was obtained from a single reconstruction time-point in early to mid-diastole and vessel contours and diameters might be different at other reconstruction time-points. Secondly, the assumptions concerning inflow and outflow may not be valid under pathologic conditions and thus leading to exaggerated mass flow in the bypass grafts. The localization of the bypasses and native coronary inlets is extracted directly from the CT scans. It is expected that a realistic inflow velocity profile would not exhibit developed flow characteristics, as in Poisseuille or Womersley theories. The main discrepancy lies on the significant presence of in plane secondary flow patterns [51] due to the motion of the coronary sinuses. Typical PC-MRI resolution does not allow for the quantification of these in-plane velocity distributions. Conversely, PC-MRI or Doppler Ultrasound can be used to quantify the blood flux discharge ratios between the first few branches of the coronary and bypass configurations. It is our intention to enhance our investigation protocol with such in-vivo measurements in order to improve the imposed outflow boundary conditions.

Moreover, both the arterial and bypass walls were simplified as being stiff. Heart movement and changing pressure at the outer wall of the vessels due to the myocardium contraction were not included in the present calculations. The importance of the large scale coronary motion induced by the beating heart is hard to quantify in CABG configurations. Although the coronary arteries move considerably throughout the cardiac cycle, it has been shown that pulsatility is the main characterizing factor of WSS distributions [52]. Additionally, the bypass grafts are intentionally fixed with surgical clips and thus experience only minor displacements near the distal anastomosis sites. Their interaction is expected to affect the pressure and WSS distributions near the anastomosis. Results might differ in elastic models of coronary arterial and CABG walls that can also take into account the compliance mismatch between the host and graft sections [53,54]. Recently, Ramaswamy et al. [44] performed a fluid dynamics analysis in a diseased section of a human coronary artery by taking into account both the large scale motion as well as compliance. This was accomplished by utilizing bi-plane angiography combined with intravascular ultrasound images. Their results showed that compliance caused substantial differences in the circumferential WSS distributions. The profound change in the distal coronary lumen dimensions during systole has major consequences as far as the outflow boundary conditions are concerned, by significantly increasing the flow resistance. Additionally, the concurrent increase of arterial pressure leads to the dilatation of the proximal coronary sections. Coronary hemodynamics and the corresponding WSS distributions will now depend on the interaction between pulsatile inflow, which maximizes during diastole, and the variation of the luminal diameter, which maximizes during systole.

Finally, the assumption that blood behaves as a Newtonian liquid must be examined further. Although it is assumed that blood exhibits shear thinning behavior in vessels of smaller diameter than the coronary arteries, there is a multitude of patient dependent parameters like the hematocrit and the intake of drugs that can alter blood viscosity. Numerical simulations of non-Newtonian flow in a two dimensional end-to-side anastomosis under pulsatile flow conditions have shown only minor effects on WSS distributions [55]. However such results are bound to depend on the particulars of the constitutive equations utilized for determining blood's viscosity.

## Conclusion

Our study has investigated the influence of patient specific geometry of end-to-side and side-to-side anastomosis on perianastomotic hemodynamics to identify geometrically driven flow features that might increase the propensity of venous graft failure. CFD analysis allowed us to differentiate hemodynamics in the two types of anastomoses and indicated significant spatial WSS variations especially in the end-to-side anastomosis configuration and an absence of stagnation areas in side-to-side anastomosis. The CFD simulations were performed in only two patients, a fact that limits the significance of our findings, which might differ in patients with different geometry. The numerical method applied herein may provide the basis for future prospective investigations correlating hemodynamic features with neointimal hyperplasia after CABG surgery.

## Competing interests

The author(s) declare that they have no competing interests.

## Authors' contributions

Guarantor of integrity of entire study: TF

Study concepts: TF, BM

Study design: TF, EB

Literature research: TF, EB, HA

Clinical studies: TS, LH, SL

Computational studies: TF, EB, DP

Data acquisition: LH, SL

Data analysis/interpretation: TF, EB, HA

Statistical analysis: TF, EB

Manuscript preparation: TF, EB

Manuscript editing: TF, EB HA, DP, BM

Manuscript revision/review: TF, EB, HA, DP, BM

All authors read and approved the final version of the manuscript.
